# The effect of environmental factors on transepithelial potential in a model Amazonian teleost, the tambaqui ( *Colossoma macropomum* ): Implications for sodium balance in harsh environments

**DOI:** 10.1111/jfb.16050

**Published:** 2025-01-09

**Authors:** Chris M. Wood, Anne Crémazy, Carolyn Morris, Ora E. Johannsson, Gudrun De Boeck, Adalberto Luis Val

**Affiliations:** ^1^ Department of Zoology University of British Columbia Vancouver British Columbia Canada; ^2^ Department of Biology McMaster University Hamilton Ontario Canada; ^3^ Department of Marine Biology and Ecology University of Miami Rosenstiel School of Marine, Atmospheric, and Earth Science Miami Florida USA; ^4^ Centre Eau Terre Environnement Institut National de la Recherche Scientifique Quebec City Québec Canada; ^5^ ECOSPHERE, Department of Biology University of Antwerp Antwerp Belgium; ^6^ Laboratory of Ecophysiology and Molecular Evolution Brazilian National Institute for Research of the Amazon (INPA) Manaus Brazil

**Keywords:** ammonia, hyperoxia, hypoxia, temperature, unidirectional sodium fluxes, water pH

## Abstract

The tambaqui (*Colossoma macropomum*, G. Cuvier 1818) thrives both in the ion‐poor waters of the Amazon and in commercial aquaculture. In both, environmental conditions can be harsh due to low ion levels, occasional high salt challenges (in aquaculture), low pH, extreme PO_2_ levels (hypoxia and hyperoxia), high PCO_2_ levels (hypercapnia), high ammonia levels (in aquaculture), and high and low temperatures. Ion transport across the gill is affected by active transport processes, passive diffusive permeability, ion concentrations (the chemical gradient), and transepithelial potential (TEP, the electrical gradient). The latter is a very important indicator of ionoregulatory status but is rarely measured. Using normoxic, normocapnic, ion‐poor, low–dissolved organic carbon (DOC) well water (27°C, pH 7.0) as the acclimation and reference condition, we first confirmed that the strongly negative TEP (−22.3 mV inside relative to the external water) is a simple diffusion potential. We then evaluated the effects on TEP of more complex waters from the Rio Negro (strong hyperpolarization) and Rio Solimões (no significant change). Additionally, we have quantified significant effects of acute, realistic changes in environmental conditions—low pH (depolarization), hypercapnia (depolarization), hypoxia (depolarization), hyperoxia (hyperpolarization), elevated NaCl concentrations (depolarization), and elevated NH_4_Cl concentrations (depolarization). The TEP responses help explain many of the changes in net Na^+^ flux rates reported in the literature. We have also shown marked effects of temperature on TEP and unidirectional Na^+^ flux rates (hyperpolarization and decreased fluxes at 21°C, depolarization and increased fluxes at 33°C) with no changes in net Na^+^ flux rates. Calculations based on the Nernst equation demonstrate the importance of the TEP changes in maintaining net Na^+^ balance.

## 1 INTRODUCTION

The net uptake of major ions, such as Na^+^, across the gills of freshwater fish is affected by (i) active transport against the concentration gradient from water to blood, (ii) passive permeability of the branchial epithelium, (iii) the concentration gradient between water and blood plasma, and (iv) the electrical gradient between water and blood plasma across the gills, which is generally termed the transepithelial potential (TEP). There is a massive literature on the first three (for detailed reviews, see Evans et al., 2005; Marshall & Grosell, 2005; Hiroi & McCormick, 2012; Hwang & Lin, 2014; Wood, 2022), but the latter has been only sparsely studied. Indeed, the only thorough review on the TEP in fish (Potts, 1984) is now 40 years old. This deficit of information is probably because the TEP is often thought to be difficult to measure, and the equipment is not widely available. In general, the TEP in freshwater fish represents a simple diffusion potential resulting from the differential permeability of the gills to cations (mainly Na^+^) versus anions (mainly Cl^−^) in the blood plasma (Potts, 1984). Theoretically, other cations (e.g., K^+^, Ca^2+^, Mg^2+^, NH_4_^+^) can also contribute to the diffusion potential. They are thought to make a negligible contribution in normal fresh water but can play an important role when elevated in the environment (e.g., Potts & Eddy, 1973; Po & Wood, 2021; Wood et al., 2020). There is only very limited evidence for an electrogenic component (i.e., resulting from charge separation by active transport) in a few freshwater species (Eddy, 1975; Potts, 1984).

A simple calculation using the Nernst equation (see Wood & Grosell, 2008) illustrates the importance of the TEP. If the TEP were to change from 0 to −25 mV in a freshwater fish (plasma [Na^+^] = 140 mmol L^−1^) living in typical fresh water (water [Na^+^] = 1 mmol L^−1^), then the electrochemical gradient driving net Na^+^ loss (or opposing net Na^+^ uptake) would be reduced by 21% from 120 to 95 mV. As a result of the logarithmic and therefore non‐linear nature of the Nernstian equation, which provides the chemical part of the electrochemical gradient, this would have the same effect on Na^+^ loss as reducing the plasma [Na^+^] from 140 to about 53 mmol L^−1^. Thus, a relatively small change in TEP can have a large effect on Na^+^ homeostasis and, therefore, the cost of ionoregulation.

Theoretically, changes in TEP would be expected to affect both active Na^+^ uptake (J^Na+^_in_) and passive Na^+^ efflux (J^Na+^_out_) across the gills. However, J^Na+^_in_ occurs by active transport for which the rate can be quickly adjusted by feeding more ATP to the pumps, increasing their affinity for Na^+^, or increasing the number of pumps that are functional, options that are not available to J^Na+^_out_. Therefore, we would predict that J^Na+^_out_ would be more responsive to TEP than J^Na+^_in_ and would be the larger contributor to changes in J^Na+^_net_ caused by alterations in TEP.

The tambaqui (*Colossoma macropomum*, G. Cuvier 1818) is of immense socioeconomic importance in aquaculture and artisanal fisheries in the Amazon basin, and it has become a model Neotropical teleost fish (reviewed by Goulding & Carvalho, 1982; Araujo‐Lima & Goulding, 1997; Prado‐Lima & Val, 2016; Nunes et al., 2017; Wood et al., 2017; Wood et al., 2018; Val & Oliveira, 2021; Amanajás & Val, 2023). In particular, the tambaqui is known for its robust physiology and ability to tolerate varying harsh environmental conditions that may occur in Amazonia. These include ion‐poor waters (in nature), elevated ion concentrations (in aquaculture), and conditions such as low pH, hypoxia, hyperoxia, hypercapnia, high environmental ammonia, and extreme temperatures (in both). These can occur in normal aquacultural practice and in the wild during the annual migration of this species between the “white waters” of the Rio Solimões and the “black waters” of the Rio Negro. They can also occur as a result of diurnal, seasonal, and climate change–related fluctuations in hydrology, temperature, and associated rates of environmental respiration, photosynthesis, and biodegradation affecting water O_2_, CO_2_, ammonia, and pH levels (Val et al., 2006; Val & Almeida‐Val, 1995; Val & Wood, 2022).

In the present study, our first goal was to test whether the TEP in tambaqui is a simple diffusion potential. Our second objective was to evaluate the acute effects of a variety of realistic environmental challenges on the TEP. These included acute exposures to Rio Negro “black water” and Rio Solimões “white water.” In view of the critical importance of water composition in determining TEP, our third objective was to document the chemical composition of these two natural waters, as well as that of the control water to which our fish were acclimated. This was well‐aerated groundwater from the campus of the Brazilian National Institute for Amazon Research (INPA) (Manaus, AM). INPA water is an ion‐poor water, similar in composition to Rio Negro water, but differs in having a much higher pH (after air equilibration) and being virtually lacking in dissolved organic carbon (DOC). Other experimental challenges included acidic pH, increases and decreases in temperature, lowered and elevated PO_2_ levels, elevated PCO_2_ levels, elevated Na^+^ concentrations, and high environmental ammonia. In all cases, the challenges tested were within the ranges reported to occur by others, or measured by us, in the tambaqui's natural or aquacultural environments. TEP measurements during all these challenges were made relative to control measurements on the same individual in its acclimation water at 27°C.

For some (but not all) of the environmental challenges, there is prior published or unpublished information on their effects on unidirectional and/or net Na^+^ flux rates in the tambaqui. These include hypoxia (Robertson et al., 2015), acidic pH (Gonzalez et al., 1998; Gonzalez et al., 2021; Wilson et al., 1999; Wood et al., 1998), and Rio Solimões and Rio Negro water (Crémazy et al., 2026; C. Morris, A. Crémazy, S. Braz‐Mota., O.E. Johannsson, C.M. Wood & A.L. Val, unpublished data). However, we are aware of no information on the effects of temperature on Na^+^ balance in this species, so our final goal was to measure unidirectional and net Na^+^ flux rates during acute low and high temperature challenges.

## 2 METHODS

### 2.1 Experimental animals

Juvenile tambaqui (*C. macropomum*) of mixed sex were obtained from an aquaculture farm (Sítio dos Rodrigues, Km 35, Rod. AM‐010, Brazil). The fish were shipped by the supplier to the Ecophysiology and Molecular Evolution Laboratory of INPA in water containing approximately 5 mmol L^−1^ NaCl, a common practice in aquaculture. They had never been in natural waters such as the Rio Negro or Rio Solimões, both of which have much lower ionic strength (Table 1). Therefore, over the first week, we progressively diluted the shipping water with INPA well water, which is ion‐poor and much closer in composition to the natural waters (Table 1). This water is available on tap in the INPA laboratory. The fish were then acclimated for another week to pure INPA water. The experiments were performed over the following 2 weeks. Note that the INPA water initially contains 3%–5% CO_2_, reflecting its groundwater origin, so we aerated it vigorously prior to use in the present study. Therefore, well‐aerated INPA water served as the control medium for all experiments. However, anecdotally, we have noticed that tambaqui survive without apparent ill effect in INPA water containing these levels of CO_2_. Acclimation and control temperature for experiments was 27°C. Fish used in the TEP experiments were 10–30 g and those used in the Na^+^ flux study were 1.5–2.2 g.

**TABLE 1:** Measured water chemistry of the three waters used in the present study prior to any experimental amendments.

	INPA water	Rio Negro	Rio Solimões
pH	7.0	4.0	6.7
DOC (mg L^−1^)	0.59	11.2	5.3
DIC (mg L^−1^)	0.30	0.21	6.63
Na^+^ (μmol L^−1^)	63	12	117
Ca^2+^ (μmol L^−1^)	5	8	697
K^+^ (μmol L^−1^)	12	6	21
Mg^2+^ (μmol L^−1^)	1	3	43
Cl^−^ (μmol L^−1^)	11	10	76
Conductivity (μS cm^−1^)	17	11	70

*Note:* Institute for Research of the Amazon (INPA) well water served as the control water in all experiments.

Abbreviations: DIC, dissolved inorganic carbon; DOC, dissolved organic carbon.

The fish were fed daily to satiation with commercial pellets (Nutripiscis‐Presence AL 45, SP Rações, São Paulo, SP, Brazil; 45% protein) but were fasted for 24–48 h prior to surgery and experiments.

### 2.2 Ethics statement

All experimental procedures were approved by the Ethics Committee on Animal Experiments of INPA under registration number 027/2015 and conformed to national animal care regulations.

### 2.3 Experimental waters

A 400‐L batch of INPA water (the same water to which the tambaqui were acclimated) was collected on June 10, 2023, from a single tap on the campus and stored with aeration at 27°C to serve as the control water throughout the study. Rio Negro water (from GPS 3° 18′ 17.7″ S 60° 20′ 26.7″ W) and Rio Solimões water (from GPS 3°18′ 26.0″ S 60° 36′ 41.9″ W) were collected in multiple 50‐L batches on May 23 and on May 26, 2023, respectively, and stored in a cool room at 12°C in the dark for approximately 2 weeks. Prior to use, these waters were warmed to 27°C and vigorously aerated for several hours, but were not filtered, so as to retain their natural composition.

### 2.4 TEP experiments

Tambaqui (10–30 g) were anaesthetized in INPA water containing 0.1 g L^−1^ MS‐222 (Syndel Laboratories, Vancouver, BC, Canada), corrected to pH 7.0 with NaOH. Indwelling intraperitoneal catheters (Clay‐Adams PE50 with PE160 sleeves, Becton, Dickinson and Co., Franklin Lakes, NJ, USA) filled with Cortland saline (Wolf, 1963) were inserted as described for toadfish by Wood and Grosell (2008). The fish were allowed to recover overnight in individual 3‐L containers (12 in total) fitted with air‐stones and served with flowing, recirculating INPA water from an aerated 400‐L reservoir.

For experimental measurements, a paired or repeated‐measures design was used. A fish was gently transferred to a smaller chamber containing 1.5 L of air‐saturated INPA water at pH 7.0, 27°C, and allowed to settle for 5 min. Three measurements of TEP were made, as described below, over 2 min, thereby providing the control data. The same fish was then transferred to the experimental medium and allowed to settle for a further 5 min, after which TEP measurements were again made in triplicate. In each case, the three measurements were averaged. In series where two experimental media were tested, the same fish was returned to the control media (INPA water) for 5 min in between the experimental tests, and again the measurements in each medium were made in triplicate. Tests showed that the order of presentation of the experimental media and control media did not matter (Table S1).

A high impedance voltmeter (Radiometer pHM84, Copenhagen, Denmark) was used to measure TEP. Agar bridges made with 3‐M KCl were connected using PE160 catheters filled with 3‐M KCl to Ag/AgCl electrodes (World Precision Instruments, Sarasoto, FL, USA), which, in turn, were connected to the voltmeter by a custom‐built dual‐electrode wire. For measurements, one bridge was connected to the external water and the other to the saline‐filled catheter in the intraperitoneal catheter of the fish. TEP was expressed relative to the apical (water) side as 0 mV after correction for the junction potential. Previous studies (Potts & Eddy, 1973; Wood & Grosell, 2008) have demonstrated that TEP measurements made in the extracellular peritoneal fluid yield identical values to those recorded from blood plasma because of the very high conductance of the extracellular fluid. Furthermore, the only external barrier epithelium of the fish with significant permeability to cations and anions is the branchial epithelium, so TEP measured from a saline‐filled catheter in the peritoneal cavity with reference to the external water as 0 mV represents the TEP across the gills.

In separate series, measurements were made after the fish were acutely exposed to the following experimental media (i.e., after the 5‐min settling period), in comparison to control measurements made on the same fish in air‐equilibrated INPA water, pH 7.0, 27°C:

Series (i): 140 mmol L^−1^ NaCl in INPA water (*N* = 6). This served to test whether the TEP was a simple diffusion potential.
Series (ii): Rio Negro water at natural pH 4.0 (Table 1), followed by Rio Solimões water at natural pH 6.7 (Table 1) (*N* = 6).
Series (iii): INPA water at pH 4.0, adjusted using HNO_3_ (*N* = 18).
Series (iv): Rio Negro water at pH 7.0, adjusted using KOH (*N* = 6).
Series (v): INPA water at a dissolved CO_2_ concentration of 1.5% CO_2_ (approximately 1.5 kPa or 11 Torr), followed by 3.0% CO_2_ (approximately 3 kPa or 22 torr) (*N* = 6).
Series (vi): INPA water at a dissolved O_2_ concentration representing 50% air saturation (approximately 10.2 kPa or 77 torr), followed by INPA water at a dissolved O_2_ concentration representing 10% air saturation (approximately 2.0 kPa or 15 torr) (*N* = 6).
Series (vii): INPA water at a dissolved O_2_ concentration representing >200% air saturation (approximately >40.9 kPa or >307 torr) (*N* = 6).
Series (viii): INPA water with an added NaCl concentration of 187 μmol L^−1^ so as to achieve a Na^+^ concentration of 250 μmol Na L^−1^, followed by INPA water with an added NaCl concentration of 2437 μmol L^−1^ so as to achieve 2500 μmol Na L^−1^ (*N* = 6). For the sake of convenience, these are termed “250 μmol L^−1^ NaCl” and “2500 μmol L^−1^ NaCl.”
Series (ix): INPA water with an added NH_4_Cl concentration of 250 μmol L^−1^, followed by INPA water with an added NH_4_Cl concentration of 2500 μmol L^−1^ (*N* = 6). Note that background total ammonia concentrations were undetectable in INPA water.
Series (x): INPA water at 21°C (*N* = 6) or INPA water at 33°C (*N* = 6). These were two different batches of fish, each with their own control measurement at 27°C.

### 2.5 Unidirectional and net Na^+^ flux rate measurements

Tambaqui (1.5–2.2 g) were placed in individual plastic chambers containing 70 mL of INPA water at 27°C (acclimation temperature, *N* = 8), 21°C (*N* = 8), or 33°C (*N* = 8) and allowed to settle for 25 min prior to the start of the flux measurements. Containers received individual aeration to ensure mixing and normoxia and were incubated in an external constant temperature bath to maintain the desired temperature. Radiolabeled ^22^Na^+^ (0.5 μCi; as NaCl, Eckert and Ziegler, Valencia, CA, USA) was then added to each chamber and allowed to mix for 5 min. Water samples (10 mL for atomic absorption analysis and 1 mL for scintillation counting) were then taken at 0 h and 1 h, after which the fish were weighed.

Unidirectional Na^+^ influx rates (J^Na+^_in_, positive), net Na^+^ flux rates (J^Na+^_net_, positive or negative), and unidirectional Na^+^ efflux rates (J^Na+^_out_, negative) in nmol g^−1^ h^−1^ were calculated as described by Wood (1992):

(1) $${J^{\mathrm{Na}}}_{\text{in}}=\frac{\left.\left({\left[R\right]}_{i}–{\left[R\right]}_{f}\right)\times V\right)}{\mathrm{SA}\times t\times M}$$

(2) $${J^{\mathrm{Na}}}_{\mathrm{net}}=\frac{\left({\left[{\mathrm{Na}}^{+}\right]}_{i}–{\left[{\mathrm{Na}}^{+}\right]}_{f}\right)\times V}{t\times M}$$

(3) $${J^{\mathrm{Na}+}}_{\text{out}}={J^{\mathrm{Na}+}}_{\mathrm{net}}-{J^{\mathrm{Na}+}}_{\text{in}}$$

where SA is the mean external specific activity (radioactivity per total Na^+^, in cpm nmol^−1^) in the water calculated from measurements of water radioactivity ([R]_i_, [R]_f_, in cpm L^−1^) and total water sodium concentration ([Na^+^]_i_, [Na^+^]_f_, in nmol L^−1^) at the start (i) and end (f) of the flux period, respectively, *t* is time (in h), *M* is body mass (in g), and *V* is the volume of the flux chamber (in L).

*Q*_10_ values (temperature coefficients) for rates over different temperature ranges were calculated in the traditional fashion (Hoar, 1983):

(4) $$Q_{10}={\left[R_{2}/R_{1}\right]}^{\left[10/\left(T_{2}-T_{1}\right)\right]}$$

where *R*_1_ is the rate at the lower temperature (*T*_1_), and *R*_2_ is the rate at the higher temperature (*T*_2_).

### 2.6 Analytical techniques for characterization of experimental media

Water samples (1 mL) for analysis of ^22^Na^+^ radioactivity were added to 5 mL of Ultima Gold AB fluor (Perkin‐Elmer, Waltham, MA, USA) and counted on a Triathler portable scintillation counter (Hidex, Helsinki, Finland). Quench was constant, so no correction was applied. Water cation concentrations (Na^+^, K^+^, Mg^2+^, Ca^2+^) were measured using a Perkin Elmer AAnalyst 800 AA spectrophotometer, Norwalk, CT, USA) in flame mode. Water Cl^−^ concentrations were measured by the colorimetric method of Zall et al. (1956), and water total ammonia concentrations were measured by the colorimetric method of Verdouw et al. (1978). Total dissolved organic carbon (DOC) and inorganic carbon (DIC) concentrations of water samples (after 0.45‐μm filtration using polyethersulfone polymer membrane hydrophilic filters [MS PES Membrane Solutions, Auburn, WA, USA]) were measured using a Model TOC‐L CSN total carbon analyser (Shimadzu, Kyoto, Japan). Water pH was recorded using a SympHony pH electrode 141 (C03243) and meter (SP70P) (VWR International, Radnor, PA, USA). Water conductivity was measured using a WTW 3310 conductivity meter (Xylem Analytics, Welheim, Germany). A gas flow mixing system (Cellasic ONIX, Millipore, Burlington, MA, USA) was used to create mixed gases to equilibrate water PO_2_ and PCO_2_ to desired levels. PO_2_ and PCO_2_ levels were checked using a DO 6+ galvanic oxygen electrode and meter (Oakton Instruments, Vernon Hills, IL, USA) and a prototype PCO_2_ micro‐optode system (PreSens Precision Sensing GmbH, Regensburg, Germany), respectively.

### 2.7 Statistics

For TEP, a paired or repeated‐measures design was used within each series, with each fish serving as its own control prior to transfer to the experimental condition(s). Therefore, the changes for each individual fish are shown in figures, together with the overall mean ± 1 SEM absolute TEP for each treatment. All data passed normality and homogeneity of variance tests, and significant differences (*p* ≤ 0.05) were detected using either Student's paired *t*‐test (two‐tailed) for paired comparisons or one‐way repeated‐measures ANOVA followed by Tukey's test for multiple comparisons. Specific comparisons of TEP changes between series employed Student's unpaired *t*‐test (two‐tailed), and one‐sample *t*‐tests were employed to determine if absolute TEP values were significantly different from 0 mV. For Na fluxes, different fish were used at each temperature, so the data are shown as bar graphs representing means ± 1 SEM, together with the individual data points. All data again passed normality and variance tests. Simple one‐way ANOVA followed by Tukey's test was used to detect significant differences (*p* ≤ 0.05).

## 3 RESULTS

### 3.1 Chemistry of experimental media

Table 1 reports the chemistry of the three waters measured at the time of the tests—that is, after they had been stored in the laboratory, warmed, and vigorously aerated. The INPA well water that was used as the control condition in all experiments was characterized by low DOC, low DIC, low inorganic ion concentrations, low conductivity, and circumneutral pH (Table 1). The Rio Negro water exhibited markedly lower pH, markedly higher DOC concentration, similar DIC concentration, and generally similar conductivity and inorganic ion levels, except for Na^+^, which was about 80% lower than in INPA water. The Rio Solimões water exhibited a pH just below neutrality, an intermediate DOC concentration, and conductivity and ion concentrations that were substantially higher than in either INPA or Rio Negro water. Ca^2+^, Mg^2+^, and DIC concentrations were particularly high, reflecting much greater hardness and alkalinity.

Conductivity was measured in all the treatments in which INPA water was modified and was very uniform (14–19 μS cm^−1^), except in those experiments where ionic compounds were tested. Thus, conductivity increased to 50 μS cm^−1^ in 250 μmol L^−1^ NH_4_Cl, 382 μS cm^−1^ in 2500 μmol L^−1^ NH_4_Cl, 42 μS cm^−1^ in 250 μmol L^−1^ NaCl, and 323 μS cm^−1^ in 2500 μmol L^−1^ NaCl. The slightly lower conductivities in the NaCl versus NH_4_Cl additions reflected the fact that slightly less total NaCl was added because of the background Na levels already in INPA water, whereas NH_4_Cl was added on top of the ions already present in INPA (Table 1). Assay of Cl^−^ and ammonia concentrations in the test waters showed that measured concentrations were within 15% of nominal concentrations.

### 3.2 Effects of environmental factors on TEP

The overall mean control TEP for tambaqui in INPA water was −22.3 ± 0.8 mV (*N* = 78), with the control group means in different series varying from −26.1 to −17.8 mV.

**FIGURE 1:**
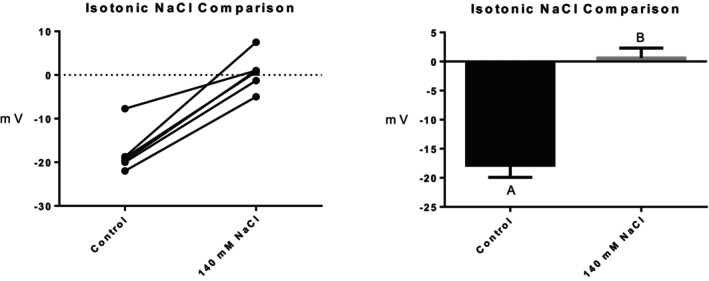
In series (i), the effect of exposure to isotonic saline NaCl in INPA water (140 mmol L^−1^) on transepithelial potential (TEP) in tambaqui. INPA water served as the control. Left panel: individual data; right panel: group means ± 1 SEM (*N* = 6). Means not sharing the same letter are significantly different (*p* ≤ 0.05). The mean TEP value in 140 mmol L^−1^ NaCl was not significantly different from 0 mV.

**FIGURE 2:**
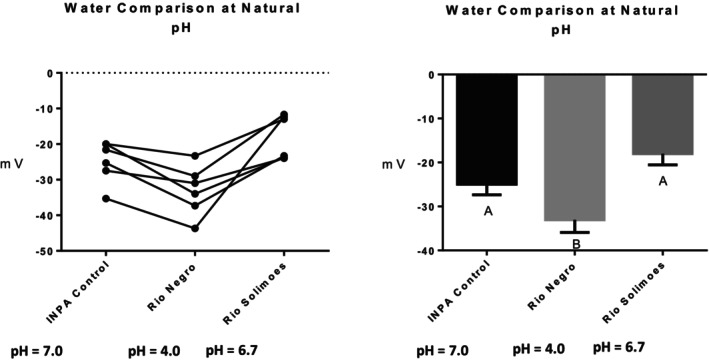
In series (ii), the effects of exposure to Rio Negro water at its natural pH 4.0 and to Rio Solimões water at its natural pH 6.7 on transepithelial potential (TEP) in tambaqui (*Colossoma macropomum*). INPA water at its natural pH 7.0 served as the control. Left panel: individual data; right panel: group means ± 1 SEM (*N* = 6). Means not sharing the same letter are significantly different (*p* ≤ 0.05).

**FIGURE 3:**
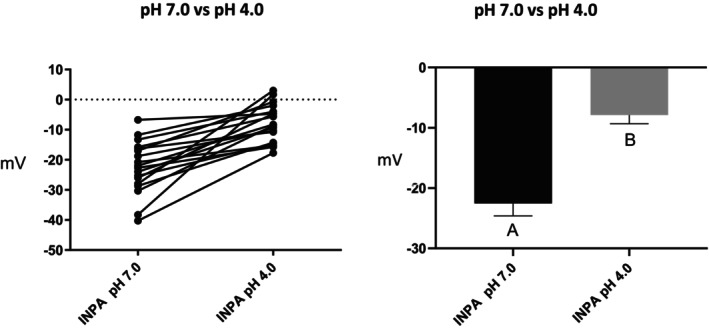
In series (iii), the effects of exposure to INPA water in which the pH was lowered from 7.0 to 4.0 using HNO_3_ on transepithelial potential (TEP) in tambaqui (*Colossoma macropomum*). INPA water at its natural pH 7.0 served as the control. Left panel: individual data; right panel: group means ± 1 SEM (*N* = 18). Means not sharing the same letter are significantly different (*p* ≤ 0.05).

**FIGURE 4:**
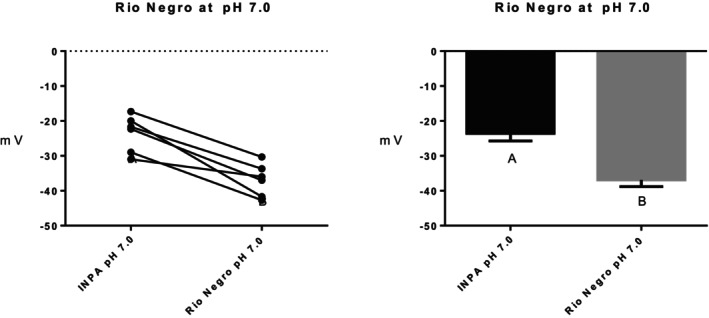
In series (iv), the effects of exposure to Rio Negro water in which the pH was increased to 7.0 using KOH on transepithelial potential (TEP) in tambaqui (*Colossoma macropomum*). INPA water at its natural pH 7.0 served as the control. Left panel: individual data; right panel: group means ± 1 SEM (*N* = 6). Means not sharing the same letter are significantly different (*p* ≤ 0.05).

**FIGURE 5:**
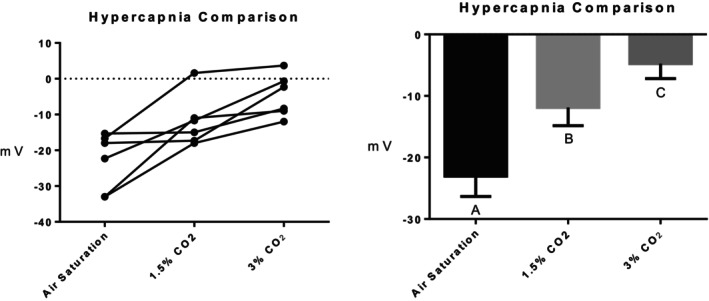
In series (v), the effects of exposure to 1.5% CO_2_ followed by 3% CO_2_ on transepithelial potential (TEP) in tambaqui (*Colossoma macropomum*). Air‐equilibrated (normocapnic, 0.04% CO_2_) INPA water served as the control. Left panel: individual data; right panel: group means ± 1 SEM (*N* = 6). Means not sharing the same letter are significantly different (*p* ≤ 0.05).

**FIGURE 6:**
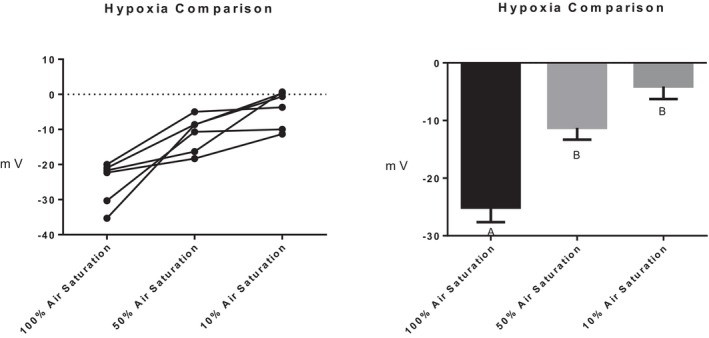
In series (vi), the effects of exposure to moderate hypoxia (50% air saturation) followed by severe hypoxia (10% air saturation) on transepithelial potential (TEP) in tambaqui (*Colossoma macropomum*). Air‐equilibrated (normoxic, 100% air saturation) INPA water served as the control. Left panel: individual data; right panel: group means ± 1 SEM (*N* = 6). Means not sharing the same letter are significantly different (*p* ≤ 0.05).

**FIGURE 7:**
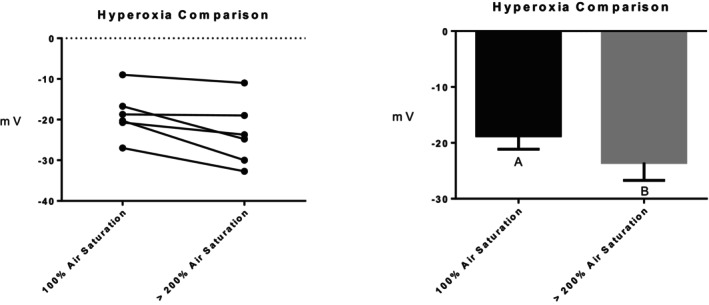
In series (vii), the effects of exposure to hyperoxia (O_2_ level > 200% air saturation) on transepithelial potential (TEP) in tambaqui (*Colossoma macropomum*). Air‐equilibrated (normoxic, 100% air saturation) INPA water served as the control. Left panel: individual data; right panel: group means ± 1 SEM (*N* = 6). Means not sharing the same letter are significantly different (*p* ≤ 0.05).

**FIGURE 8:**
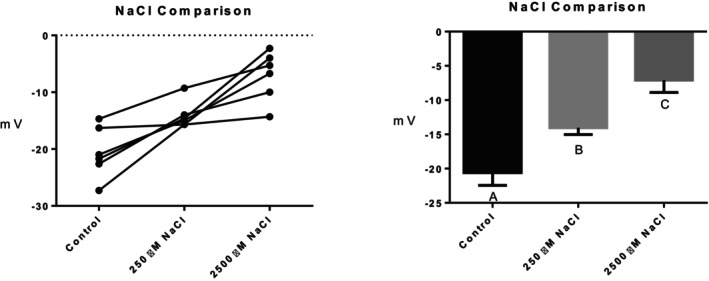
In series (viii), the effects of exposure to INPA water, first with 250 μmol L^−1^ NaCl and then with 2500 μmol L^−1^ NaCl, on transepithelial potential (TEP) in tambaqui (*Colossoma macropomum*). INPA water without added NaCl served as the control. Left panel: individual data; right panel: group means ± 1 SEM (*N* = 6). Means not sharing the same letter are significantly different (*p* ≤ 0.05).

**FIGURE 9:**
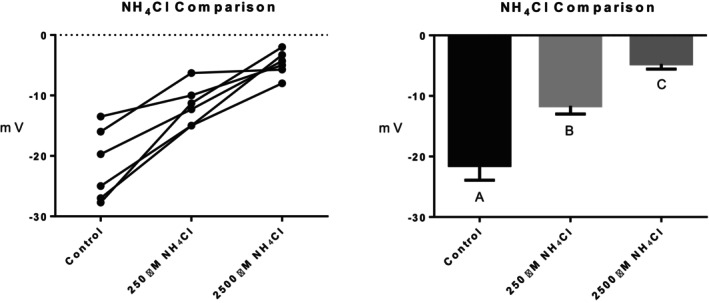
In series (ix), the effects of exposure to INPA water, first with 250 μmol L^−1^ NH_4_Cl and then with 2500 μmol L^−1^ NH_4_Cl, on transepithelial potential (TEP) in tambaqui (*Colossoma macropomum*). INPA water without added NH_4_Cl served as the control. Left panel: individual data; right panel: group means ± 1 SEM (*N* = 6). Means not sharing the same letter are significantly different (*p* ≤ 0.05).

**FIGURE 10:**
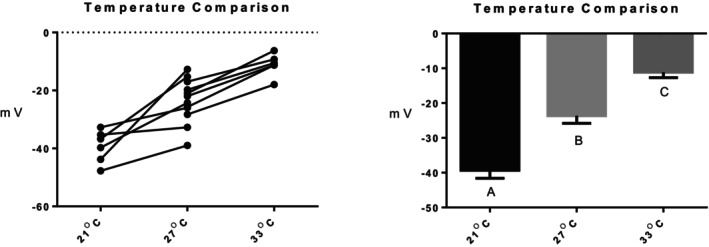
In series (x), the effects of acute exposure to 21°C (*N* = 6) or 33°C (*N* = 6) on transepithelial potential (TEP) in tambaqui (*Colossoma macropomum*). INPA water at the acclimation temperature of 27°C (*N* = 12) served as the control. Left panel: individual data; right panel: group means ± 1 SEM. Means not sharing the same letter are significantly different (*p* ≤ 0.05).

Series (i) 140 mM NaCl: Exposure of tambaqui to isotonic NaCl raised the TEP to a value not significantly different from 0 mV (Figure 1), indicating that it is probably a simple diffusion potential.
Series (ii) River comparison: Exposure of tambaqui to Rio Negro water at its natural pH 4.0 significantly lowered (i.e., hyperpolarized) the TEP by a further −8 mV relative to the INPA control. In contrast, exposure to Rio Solimões water at its natural pH 6.7 raised (i.e., depolarized) the TEP by 6 mV relative to the control, though the latter change was not significant (*p* = 0.076). However, the mean TEP values in Rio Negro (−33.1 ± 2.9 mV, *N* = 6) and Rio Solimões waters (−18.0 ± 2.5 mV, *N* = 6) were significantly different from one another (Figure 2).
Series (iii) INPA water at pH 4.0: When tambaqui were exposed to INPA water in which the pH was lowered from 7.0 to 4.0 with HNO_3_, the TEP was strongly depolarized, rising by almost 15 mV (Figure 3). However, the mean value (−7.9 ± 1.5 mV, *N* = 18) remained significantly below 0 mV (*p* < 0.0001).
Series (iv) Rio Negro water at pH 7.0: Rio Negro water was tested with its pH raised to 7.0 with KOH (Figure 4). The response, relative to the INPA control, was a strong, significant, hyperpolarization of about 13 mV.
Series (v) Hypercapnia: Exposure of tambaqui to 1.5% CO_2_ in INPA water depolarized TEP by about 11.5 mV and by about 18.5 mV when the CO_2_ level was raised to 3% (Figure 5). These responses were significantly different from the control and from each other. At the higher CO_2_ level, the TEP was not significantly different from 0 mV. Notably, the pH of the water dropped from the control level of 7.0 to 4.2 at 1.5% CO_2_ and to 4.0 at 3% CO_2_.
Series (vi) Hypoxia: Exposure of tambaqui to moderate hypoxia (50% air saturation) in INPA water resulted in a depolarization of about 14 mV, and this increased to 21 mV in severe hypoxia (10% air saturation) (Figure 6). Both changes were significantly different from the control value but not from each other. The TEP value at 10% air saturation was not significantly different from 0 mV.
Series (vii) Hyperoxia: When tambaqui were exposed to an O_2_ level in INPA water that was raised to >200% air saturation, there was a modest, but significant, hyperpolarization of the TEP by about 5 mV (Figure 7).
Series (viii) Elevated NaCl: Exposure of tambaqui to INPA water, first with 250 μmol L^−1^ NaCl and then with 2500 μmol L^−1^ NaCl, resulted in progressive depolarizations of TEP by 6.5 and 13.5 mV, respectively (Figure 8). Both changes were significantly different from each other and from the control.
Series (ix) Elevated NH_4_Cl: Exposure of tambaqui to INPA water, first with 250 μmol L^−1^ NH_4_Cl and then with 2500 μmol L^−1^ NH_4_Cl, resulted in a similar pattern of TEP depolarization (about 9.5 and 16.5 mV, respectively; Figure 9), as observed with the same concentrations of NaCl in series (viii) (Figure 8). Again, both changes were significantly different from each other and from the control.
Series (x) Temperature: Relative to the acclimation temperature of 27°C, acute exposure of tambaqui to 21°C in INPA water hyperpolarized TEP by about −15.5 mV, whereas acute exposure to 33°C depolarized TEP by about 12.5 mV (Figure 10). The absolute values at the three temperatures were significantly different from one another.

### 3.3 Effects of temperature on unidirectional and net Na^+^ fluxes

**FIGURE 11:**
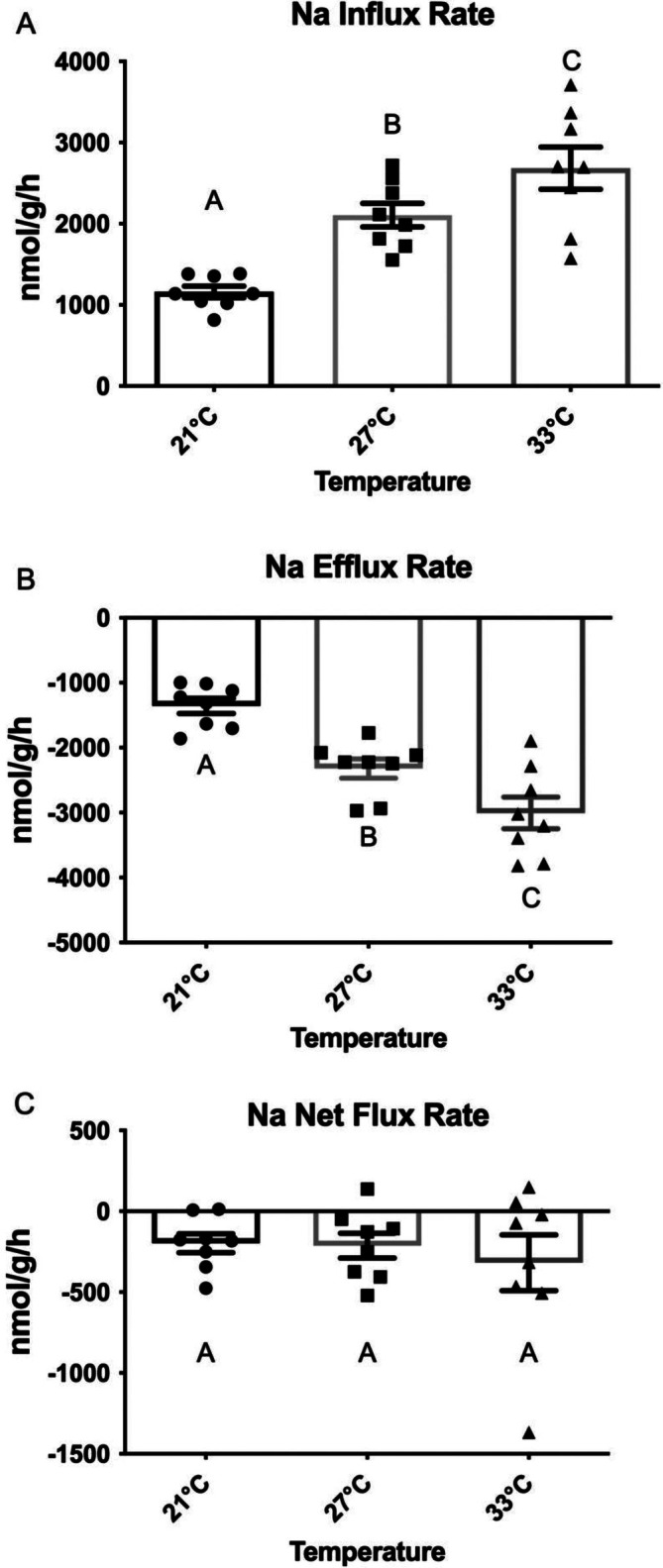
The effects of acute exposure to 21°C (*N* = 8) or 33°C (*N* = 8) on Na flux rates in tambaqui (*Colossoma macropomum*). INPA water at the acclimation temperature of 27°C (*N* = 8) served as the control. (a) unidirectional Na influx rates (J^Na^_in_); (b) unidirectional Na efflux rates (J^Na^_out_); and (c) net Na flux rates (J^Na^_net_). Means ± 1 SEM with individual data points shown. Means not sharing the same letter are significantly different (*p* ≤ 0.05).

At all three temperatures, unidirectional Na^+^ flux rates (J^Na+^_in_ and J^Na+^_out_) of tambaqui in INPA water were manyfold (6–10 x) greater than net Na^+^ flux rates (J^Na^_net_). At the acclimation temperature of 27°C, the fish were in approximate Na^+^ balance, with J^Na+^_in_ (Figure 11a) and J^Na+^_out_ (Figure 11b) values being almost equal to one another, such that J^Na+^_net_ values (Figure 11c) were just slightly negative (*p* = 0.03 relative to 0 nmol g^−1^ h^−1^). Although there were large alterations in J^Na+^_in_ and J^Na+^_out_ with acute temperature changes, J^Na+^_net_ values stayed slightly negative but close to 0 nmol g^−1^ h^−1^ (*p* = 0.01 at 21°C and *p* = 0.09 at 33°C).

An acute temperature reduction to 21°C resulted in 55%–60% decrease in both J^Na+^_in_ and J^Na+^_out_, whereas an acute temperature increase to 33°C resulted in 25% increase in both J^Na+^_in_ and J^Na+^_out_. All of these changes were significant, whereas J^Na+^_net_ did not vary significantly across temperatures. *Q*_10_ values calculated for J^Na+^_in_ and J^Na+^_out_ from these mean data (Table 2) indicate a greater temperature dependency between 21 and 27°C than between 27 and 33°C.

**TABLE 2:** *Q*_10_ values for Na influx (J^Na^_in_), efflux (J^Na^_out_), and net flux (J^Na^_net_) rates of tambaqui (*Colossoma macropomum*) in INPA water in series (x) and (xi), calculated from the mean data in Figure 11.

	21–27°C	27–33°C	21–33°C
J^Na^_in_	2.70	1.50	2.01
J^Na^_out_	2.44	1.54	1.94
J^Na^_net_	1.13	1.96	1.49

*Note:* Tambaqui were acclimated to 27°C and then acutely transferred to either 21 or 33°C.

## 4 DISCUSSION

### 4.1 The nature of the TEP in the tambaqui

The mean absolute TEP (−22.3 mV) of the tambaqui in INPA water was typical of other teleosts in similarly diluted fresh water (Po & Wood, 2021; Potts, 1984). It is also consistent with three previous measurements for this species in similar water quality (−17 to −23 mV) reported by Wood et al. (1998), Wood et al. (2017), and Sadauskas‐Henrique et al. (2019). The abolition of the TEP by exposure to 140 mmol L^−1^ NaCl (Figure 1) in series (i) was similar to previous findings in the fathead minnow (Wood et al., 2020), rainbow trout, and goldfish (Po & Wood, 2021), and provides classic evidence (Potts, 1984) for the absence of an electrogenic component to the TEP in the tambaqui. If an electrogenic component were present, it would have persisted for some time when the concentration gradients across the gills for the two major strong ions in the blood plasma (Na^+^ and Cl^−^) were eliminated by exposure to isotonic saline. This did not occur. We can conclude that the negative TEP (inside) is a simple diffusion potential resulting from the differential passive permeabilities (*P*) of the gill epithelium to strong cations versus strong anions (i.e., *P*_Na+_ > *P*_Cl−_) as in most other freshwater fish (Potts, 1984). In turn, this means that the effects of the various environmental factors tested in this study can be interpreted as resulting from either a change in the permeability ratio or a change in the ionic gradients.

### 4.2 The effect of pH on TEP in INPA water

The marked depolarization of TEP observed when tambaqui were exposed to pH 4.0 in INPA water (Figure 3) in series (iii) agreed exactly with the depolarization reported by Sadauskas‐Henrique et al. (2019) when the same low pH treatment (pH 4.0 using HNO_3_) was administered in similar water quality. In both studies, the TEP increased by 15 mV. However, there was a large quantitative difference with the results of Wood et al. (1998), who reported that acute exposure to pH 4.0 caused a depolarization of about 41 mV in the tambaqui that they analysed, raising the TEP to a significantly positive value of +18 mV. The reason for this difference is unknown. It may reflect the fact that Wood et al. (1998) used H_2_SO_4_ rather than HNO_3_ to lower pH, and/or that the tambaqui studied by Wood et al. (1998) were approximately 10‐fold larger in body size. Interestingly, McWilliams and Potts (1978), using brown trout of intermediate size (threefold larger) and acidification with H_2_SO_4_, reported an intermediate depolarization of about 22 mV at pH 4.0. McWilliams and Potts (1978) interpreted the depolarization response to a high gill *P*_H+_ resulting in H^+^ ion entry into the fish, but based this conclusion on calculations assuming that *P*_Cl−_ remained unchanged at low pH. We now know that this is not true for both trout (Wood, 1989) and tambaqui (Wilson et al., 1999; Wood et al., 1998): Cl^−^ losses increase at pH 4.0 in both species, net acid uptake does not occur, and internal acid–base disturbance is negligible. A more likely explanation is that low pH, while increasing absolute permeability of the gills, lowers the *P*_Na+_/*P*_Cl_^−^ ratio, thereby driving the TEP less negative or more positive.

As part of a different investigation on this same batch of tambaqui, C. Morris, A. Crémazy, S. Braz‐Mota., O.E. Johannsson, C.M. Wood & A.L. Val, (unpublished data) found that net Na^+^ flux rates became highly negative after they were transferred from INPA water at pH 7.0 to INPA water at pH 4.0 (HNO_3_), as in the present TEP study. This is exactly the result expected from the depolarization of TEP (Figure 3). It also agrees with earlier studies using H_2_SO_4_ that showed a switch to elevated Na^+^ loss rates when tambaqui were acutely exposed to pH 4.0 (Wilson et al., 1999) or pH 3.5 in INPA water (Gonzalez et al., 1998; Wood et al., 1998). Indeed, of five characiform fishes tested, tambaqui exhibited the greatest net Na^+^ loss rates at low pH (Gonzalez et al., 1998). In marked contrast, Gonzalez et al. (2021) reported that acute exposure of tambaqui to either pH 4.5 or pH 3.5 did not affect net Na^+^ flux rates, which remained positive. As Gonzalez et al. (2021) used HNO_3_, fish of a similar size to those studied by C. Morris, A. Crémazy, S. Braz‐Mota., O.E. Johannsson, C.M. Wood & A.L. Val, (unpublished data) and the present study, as well as similar INPA water, but did not measure TEP, the difference cannot be explained at present. Regardless, it is clear that during continued exposure, Na^+^ balance recovers (Wilson et al., 1999; Wood et al., 1998), and tambaqui can survive indefinitely at pH 4.0 (Aride et al., 2007). In future studies, it would be informative to measure TEP during acclimation to pH 4.0.

### 4.3 The effects of pH on TEP in Rio Negro water

In series (ii), acute exposure to Rio Negro water at its native pH 4.0 caused a hyperpolarization, making TEP more negative by 8 mV (Figure 2). We would predict that this should help Na^+^ retention, and this is exactly what happened in this same batch of tambaqui where net Na^+^ flux rate switched from negative to zero (C. Morris, A. Crémazy, S. Braz‐Mota., O.E. Johannsson, C.M. Wood & A.L. Val, unpublished data). These responses are remarkable, inasmuch as pH 4.0 exposure in INPA water (series iii) markedly depolarized TEP (Figure 3) and induced net Na^+^ loss (C. Morris, A. Crémazy, S. Braz‐Mota., O.E. Johannsson, C.M. Wood & A.L. Val, unpublished data). The major difference between the two waters is the much larger DOC component in the Rio Negro, which is the cause of the low pH (Kullberg et al., 1993; Thurman, 1985), in contrast to the added HNO_3_ in INPA water. Indeed, there is abundant evidence that optically dark allochthonous DOC, such as that in the Rio Negro (Thurman, 1985; Val & Wood, 2022), causes TEP to become more negative and protects against Na^+^ loss at acidic pH (reviewed by Galvez et al., 2008; Wood et al., 2011; Morris et al., 2021; Val & Wood, 2022). Working in INPA water, Sadauskas‐Henrique et al. (2019) added a DOC extract collected from an upstream site on the Rio Negro and found that it prevented the depolarization caused by pH 4.0 but did not induce hyperpolarization. Furthermore, it accelerated Na^+^ loss rate at pH 4.0. However, their experiment was different, as the background water was not Rio Negro, acidification was caused by added HNO_3_, and the DOC extract had been stored for several years.

In series (iv), exposure of tambaqui to Rio Negro water with its pH raised to 7.0 with KOH again caused a strong hyperpolarization (Figure 4). The absolute mean TEP (−36.9 ± 1.9 mV, *N* = 6) was more negative but not significantly different (*p* = 0.30) from that observed in series (ii) when Rio Negro water was tested at its native pH 4.0 (−33.1 ± 2.9 mV, *N* = 6; Figure 2). Furthermore, the extent of the hyperpolarization at pH 4.0 relative to the paired control at pH 7.0 (−13.3 ± 2.2 mV mV) was greater but not significantly different (*p* = 0.10) from that observed in series (ii) at pH 4.0 (−8.1 ± 1.8 mV, *N* = 6). Again, we attribute the effects to the presence of native allochthonous DOC in Rio Negro water. Sadauskas‐Henrique et al. (2019) reported similar but weaker responses with the aged Rio Negro DOC extract mentioned earlier. Therefore, we can conclude that the effect of Rio Negro water on TEP appears to be largely independent of pH. Nevertheless, net Na^+^ flux rates became significantly positive in this same batch of tambaqui exposed to Rio Negro water at pH 7.0, in contrast to the net balance situation seen with Rio Negro at pH 4.0 (C. Morris, A. Crémazy, S. Braz‐Mota., O.E. Johannsson, C.M. Wood & A.L. Val, unpublished data).

### 4.4 TEP in Rio Solimões water

Acute exposure to Rio Solimões water at its native pH (6.7) in series (ii) caused a modest but non‐significant depolarization of TEP by about 6 mV relative to INPA water at pH 7.0 (Figure 2). Despite the similar pHs, the Rio Solimões has a very different chemistry, particularly much higher DOC, Na^+^, Ca^2+^, and Mg^2+^ concentrations, compared to INPA water (Table 1). The elevated DOC would be expected to hyperpolarize the TEP as explained earlier, though Sadauskas‐Henrique et al. (2025) found that Rio Solimões DOC (added to the water as an extract) was much less potent in this regard than Rio Negro DOC added in the same manner (Sadauskas‐Henrique et al., 2019). On the contrary, elevated Na^+^, Ca^2+^, and Mg^2+^ would be expected to depolarize the TEP (McWilliams & Potts, 1978; Morris et al., 2021; Wood et al., 1998; Wood et al., 2020). Therefore, the modest TEP response likely reflects these opposing factors. Overall, these factors would be expected to reduce net Na^+^ efflux rates, as well as promote active Na^+^ uptake (Val & Wood, 2022; Wood et al., 2011). This likely explains the shift to a strongly positive net Na^+^ balance observed in this same batch of tambaqui acutely exposed to Rio Solimões water (Crémazy et al., 2026; C. Morris, A. Crémazy, S. Braz‐Mota., O.E. Johannsson, C.M. Wood & A.L. Val, unpublished data) and in a different batch exposed to Rio Solimões extract (Sadauskas‐Henrique et al., 2025). Tambaqui maintained similar unidirectional Na^+^ influx and efflux rates and net Na^+^ balance in both Rio Solimões water and Rio Negro water, whereas another recent study (G. De Boeck, personal communication) reported positive Na^+^ balance for tambaqui in both waters.

### 4.5 The effects of hypercapnia on TEP in INPA water

We are aware of no previous studies of hypercapnia on TEP in any fish and no data on Na^+^ balance in tambaqui at high PCO_2_. The PCO_2_ levels (1.5% and 3%) used in series (v) may seem unusually high to most fish physiologists, but they are routine in the Amazon basin where waters are strongly supersaturated with CO_2_, owing to the intense respiration and photooxidation of organic materials and limited outgassing (reviewed by Richey et al., 2002; Johannsson et al., 2017; Val & Wood, 2022). In the poorly buffered INPA water, 1.5% and 3% CO_2_ lowered water pH to 4.2 and 4.0, respectively. The responses were strong depolarization of the TEP (Figure 5). From the present data, it is not possible to determine whether the responses to high PCO_2_ were simply responses to the low water pH or involved an additional mechanism. For example, at 3% CO_2_, the absolute TEP was −4.8 ± 2.4 mV (*N* = 6), whereas at this same pH 4.0 using HNO_3_ under normocapnia, the TEP was not significantly different (*p* = 0.30) at −7.9 ± 1.5 mV (*N* = 18). The same was true (*p* = 0.26) for the extent of the depolarization (18.3 ± 2.5 mV, *N* = 6 vs. 13.2 ± 2.4 mV, *N* = 18). In future studies, the use of buffers may illuminate this issue on a mechanistic level but would not be environmentally realistic. More valuable will be measurements of unidirectional and/or net Na^+^ flux rates in tambaqui exposed to high PCO_2_ and studies on TEP and flux rates under combined hypercapnia and hypoxia, as the two stressors usually co‐occur in nature.

### 4.6 The effects of hypoxia and hyperoxia on TEP in INPA water

The hypoxic and hyperoxic PO_2_ levels (50%, 10%, and >200% air saturation) used in series (vi) and (vii) were also very realistic for the Amazon basin (Val et al., 2006; Val & Almeida‐Val, 1995). Indeed, intermittent hypoxia due to respiration, interspersed with normoxia or hyperoxia due to photosynthesis, is one of the most important ecological and evolutionary drivers for Amazon fishes, and most show physiological and/or structural adaptations to low water PO_2_ (Val & Wood, 2022). For example, the tambaqui grows an extended lower lip after a few hours in hypoxia to facilitate skimming of the O_2_‐rich surface layer of the water (Rantin & Kalinin, 1996; Val & Oliveira, 2021), though this did not occur in the very acute time scale of our experiments. Hypoxia at both 50% and 10% air saturation caused significant depolarization of the TEP (Figure 6), responses that were quantitatively almost identical to those caused by 1.5% and 3% CO_2_ in series (v) (Figure 5). As mentioned earlier, in future studies, it will be instructive to look at the combined effects of hypoxia and hypercapnia.

There appear to be only two previous investigations of TEP during hypoxia—in the Amazonian oscar (Wood et al., 2009) and the Atlantic killifish in fresh water (Wood & Grosell, 2015). Like the tambaqui, these species are extremely hypoxia‐tolerant and show the unusual type of osmorespiratory compromise in which gill permeability to many substances, including Na^+^, is reduced during hypoxia (reviewed by Wood & Eom, 2021). Both active Na^+^ uptake and passive diffusive losses are reduced, so that net Na^+^ fluxes remain more or less unchanged (tambaqui—Robertson et al., 2015; oscar—Wood et al., 2007; Wood et al., 2009; killifish—Giacomin et al., 2020). The TEP depolarization at 10% air saturation in the tambaqui (Figure 6) was very similar to that in the oscar at this same PO_2_, but in the killifish, exactly the opposite occurred, a shift to a negative TEP. However, the killifish is euryhaline, maintains an unusual positive TEP in fresh water, and has markedly different gill ionoregulatory mechanisms from most freshwater fish (Giacomin et al., 2020; Wood & Grosell, 2008). Regardless, in all three species, there is evidence for general membrane channel closure during hypoxia, and this likely reduces absolute permeability and, therefore, Na^+^ losses, though the effects on the *P*_Na+_/*P*_Cl−_ ratio may differ among species, reflected in the different TEP responses. In the tambaqui, hyperoxia moderately hyperpolarized the TEP (Figure 7) in series (vii), suggesting that the PO_2_‐dependent trend in *P*_Na+_/*P*_Cl_− continues during O_2_ supersaturation, presumably helping Na^+^ retention when the channels are fully open. On the contrary, the only previous study on hyperoxia that we are aware of reported a very slight depolarization of TEP in the euryhaline rainbow trout in fresh water (Wood, 1991). Again, this suggests that TEP responses to changes in environmental PO_2_ are species‐dependent.

### 4.7 The effects of elevated NaCl in INPA water

The lower NaCl concentration (250 μmol L^−1^) used in series (viii) is rarely exceeded in natural Amazonian waters (e.g., Table 1), but the higher concentration (2500 μmol L^−1^) is routinely exceeded in tambaqui aquaculture (see Methods). The concentration‐dependent depolarization by NaCl (Figures 1 and 8) has now been observed in many freshwater fish (Po & Wood, 2021; Wood et al., 2020) and provides classic evidence that the TEP is a diffusion potential (Potts, 1984; Wood et al., 2020). The progressively more positive TEP occurs because the outward diffusion gradient for Na^+^ is reduced, and this in itself helps limit Na^+^ uptake until the TEP reaches 0 mV when the gradient is eliminated (Figure 1).

### 4.8 The effects of elevated NH_4_Cl in INPA water

Both the lower (250 μmol L^−1^) and higher (2500 μmol L^−1^) NH_4_Cl concentrations tested in series (ix) (Figure 9) can occur in the crowded conditions of aquaculture, and tambaqui can excrete ammonia against the latter concentration (Wood et al., 2017), which is well below their 96‐h LC_50_ of 7800 μmol L^−1^ at pH 7.0 (Souza‐Bastos et al., 2017). Indeed, TEP was measured by Wood et al. (2017) in tambaqui exposed to 2500 μmol L^−1^ NH_4_Cl, and a depolarization of about 9 mV and continued ammonia excretion were both maintained for 58 h. This is lower than the depolarization (about 16.5 mV) seen at this concentration in the present study (Figure 9), but the present fish were 10‐fold smaller. Qualitatively similar TEP and ammonia excretion responses have been observed in response to elevated water NH_4_HCO_3_ in trout, goldfish, and carp (Liew et al., 2013; Wood & Nawata, 2011). In all three, the NH_4_HCO_3_‐induced depolarizations were associated with increased Na^+^ efflux rates and more negative net flux rates of Na^+^, as would be predicted. Wright and Wood (2012) pointed out that the depolarization could be an important response to limit NH_4_^+^ uptake and improve the electrochemical gradient for ammonia excretion—that is, the fish is sacrificing Na^+^ balance, at least temporarily, for waste nitrogen homeostasis. The concentration‐dependent depolarizations caused by external NH_4_Cl (Figure 9) were very similar to those caused by external NaCl in series (viii) (Figure 8). This represents a case where a cation (i.e., NH_4_^+^) other than Na^+^ can make an important contribution to the diffusion potential, as noted in the Introduction. In future it would be of interest to measure both Na^+^ and ammonia flux rates in tambaqui exposed to high environmental ammonia.

### 4.9 Effects of temperature on TEP and unidirectional and net Na^+^ fluxes

The temperatures employed in series (x) (Figure 10) and in the unidirectional Na^+^ flux measurements (Figure 11) were environmentally realistic. Tambaqui are eurythermal, inhabiting natural waters with temperatures ranging from 40 to 22°C, and in aquaculture down to 14°C; their optimal temperature appears to be around 27°C (Amanajás & Val, 2023; Merola & Pagán‐Font, 1988). TEP exhibited a strong temperature dependency, with substantial hyperpolarization at 21°C and depolarization at 33°C (Figure 10), a pattern that was paralleled by decreases in both J^Na+^_in_ (Figure 11a) and J^Na+^_out_ (Figure 11b) at low temperature and increases in both at high temperature, with J^Na+^_net_ remaining unchanged (Figure 11c). The *Q*_10_ values for both unidirectional flux rates (Table 2) were virtually identical and typical of other biological processes, such as O_2_ consumption in tambaqui (Saint‐Paul, 1984; Wood et al., 2017) and other fish (Crawshaw, 1979; Onukwufor & Wood, 2020; Roberts, 1964; Walsh et al., 1983), with lower *Q*_10_ values in the higher temperature range (27–33°C), and overall values around 2.0. Similarly, studies on other species (Evans, 1969; Motais & Isaia, 1972; Onukwufor & Wood, 2020) have reported that temperature has minimal effects on J^Na+^_net_.

It is surprising, in light of the current interest in temperature, that there appear to be no previous studies on the effects of temperature on TEP, and only two studies report on the effects of temperature on unidirectional Na^+^ flux rates in freshwater fish. Maetz (1972) acutely exposed goldfish, acclimated to 16°C, to 6°C and reported greater reduction in J^Na+^_in_ (*Q*_10_ = 3.0) than J^Na+^_out_ (*Q*_10_ = 1.7), such that J^Na+^_net_ became highly negative at the lower temperature. Gonzalez and McDonald (2000) reported that J^Na+^_in_ was markedly reduced when trout (overall *Q*_10_ ~ 3.6) and shiner (overall *Q*_10_ ~ 2.1) acclimated to 15°C were challenged with 10 and 5°C; J^Na+^_out_ and J^Na+^_net_ were not measured. There is a clear need for additional studies on more species, focusing on the simultaneous effects of temperature on TEP, J^Na+^_in_, J^Na+^_out_, and J^Na+^_net_ before systematic conclusions can be drawn. One note of caution with respect to the present results is that the tambaqui used for TEP measurements were about 10 times larger than those used for unidirectional Na^+^ flux measurements (see Methods). To our knowledge, no previous studies have investigated the effects of body size on these parameters in tambaqui.

Nevertheless, in tambaqui, it is likely that TEP played an important role in the J^Na^_out_ and J^Na^_net_ responses, and indirectly in the J^Na^_in_. Using the Nernst equation and calculations analogous to those in the Introduction (see Wood & Grosell, 2008), the measured external Na^+^ concentration in INPA water (Table 1) and an assumed plasma Na^+^ concentration of 158 mmol L^−1^ (Sadauskas‐Henrique et al., 2019; Wood et al., 1998), the TEP component reduced the electrochemical gradient, driving net Na^+^ loss by 6% at 33°C, 12% at 27°C, and 21% at 21°C. Although these may seem like relatively modest contributions, they are equivalent to reducing plasma Na^+^ concentration from 158 mmol L^−1^ to 104, 63, and 33 mmol L^−1^ at 33, 27, and 21°C, respectively, reflecting the logarithmic and therefore non‐linear nature of the Nernst equation (see Introduction). Clearly, if these changing TEP components were not present, then a substantial amount of ATP energy would have to be funneled into the active pumps responsible for J^Na^_in_, so as to maintain the plasma Na^+^ level at 158 mmol L^−1^. As for PO_2_ and pH, the effects of temperature on TEP are probably mediated by alterations in the *P*_Na+_/*P*_Cl−_ ratio, as it occurs in simplified phospholipid membrane systems (Papahadjopoulos et al., 1972).

### 4.10 Concluding remarks

Throughout, we have attributed TEP changes to alterations in *P*_Na+_/*P*_Cl−_ ratio and/or ionic gradients changing the diffusion potential across the gill epithelium. However, the gill epithelium comprises a heterogeneous surface. For example, we know from studies on the osmorespiratory compromise (reviewed by Wood & Eom, 2021) that, as environmental PO_2_ changes, different regions may receive more or less blood flow, diffusion distances may change as pillar cells in the respiratory lamellae contract or relax, and indeed some lamellae may be shut down entirely. Pavement cells may quickly cover or uncover ionocytes, therefore, effectively closing off or opening up ion channels. The recorded TEP represents the mean value for the entire gill in the same way that ion flux rate measurements represent mean values for the entire gill. It is quite possible that morphofunctional alterations in the relative proportions of the gill surface devoted to different processes may result in changes in the mean *P*_Na+_/*P*_Cl−_ ratio or mean ionic gradients, thereby altering the mean TEP. This is a rich area for future investigation.

The gill is a multitasking organ capable of complex responses to environmental challenges. Clearly every one of the challenges tested here, which may be routine occurrences in the harsh environment of the tambaqui, had marked and immediate effects on its TEP and, therefore, marked effects on the electrochemical gradient across the gill. Some of these changes were favorable for Na^+^ homeostasis, and others unfavorable, thereby decreasing or increasing ionoregulatory costs, respectively. These immediate responses in TEP will be occurring continuously, as the fish navigates the variability and gradients in its natural environment. In the present study, each of the challenges has been studied in isolation. An important future goal will be to understand how these interact with one another in altering TEP, and to tease out the relative influences of these factors in natural black, white, and clear waters of the Amazon.

Perhaps the most important conclusion that can be drawn from this investigation is that if we are to fully understand the ionoregulatory function of the gill in dynamic environments, it is not enough to simply measure plasma and water ion concentrations, active transport mechanisms, and passive permeability processes. The TEP is also a key player, but it has been rarely taken into account. Indeed, despite the foundational work of W.T.W. Potts (e.g., Potts, 1984), the electrical physiology of the gill has been sadly neglected. We hope that the present study will inspire others to give the TEP the attention that it deserves.

## AUTHOR CONTRIBUTIONS

Chris M. Wood, Anne Crémazy, and Carolyn Morris conceived the study. Chris M. Wood, Anne Crémazy, and Carolyn Morris performed the experiments and analysed the data. Carolyn Morris, Anne Crémazy, Ora E. Johannsson, and Gudrun De Boeck collected the natural waters and chemically characterized them. Adalberto Luis Val managed logistics and provided overall supervision. Chris M. Wood wrote the first draft, and all authors edited it.

## FUNDING INFORMATION

Supported by an NSERC (Canada) Discovery Grant (RGPIN‐2023‐03714) to Chris M. Wood. Anne Crémazy was supported by an NSERC (Canada) Discovery Grant (RGPIN‐2019‐04400). This study was partially funded by CNPq (Brazilian National Research Council), CAPES (Coordination of Superior Level Staff Improvement), and FAPEAM (Amazonas State Research Foundation) via funding for INCT ADAPTA (CNPQ process no. 465540/2014‐7, CAPES—finance code 001, and FAPEAM process 062.01187/2017) to Adalberto Luis Val. Adalberto Luis Val is the recipient of a research fellowship from CNPq. Chris M. Wood and Gudrun De Boeck received ADAPTA fellowships.

## Supporting information


**Data S1.** Supporting information.
