# Supplementary material for: The effect of environmental factors on transepithelial potential in a model Amazonian teleost, the tambaqui ( Colossoma macropomum ): Implications for sodium balance in harsh environments
Source: J Fish Biol. 2025 Jan 9;108(3):906–21. doi: 10.1111/jfb.16050 (PMC13122572; doi:10.1111/jfb.16050)
Supplement: Supplementary file 1 — Data S1. Supporting information. [file JFB-108-906-s001.docx]

**Supplementary Information**

**The Effect of Environmental Factors on Transepithelial Potential in a Model Amazonian Teleost, the Tambaqui *(Colossoma macropomum*): Implications for Sodium Balance in Harsh Environments**

Chris M. Wood^1,2,3^, Anne Crémazy^4^, Carolyn Morris^1^, Ora E. Johannsson^1^, Gudrun De Boeck^5^, and Adalberto Luis Val^6^

^1^Dept. of Zoology, University of British Columbia, Vancouver, BC, Canada V6T 1Z4

^2^Dept. of Biology, McMaster University, Hamilton, ON, Canada L8S 4K1

^3^Dept. of Marine Biology and Ecology, University of Miami Rosenstiel School of Marine, Atmospheric, and Earth Science, Miami, FL, USA

^4^Centre Eau Terre Environnement, Institut National de la Recherche Scientifique, Québec, QC, Canada G1K 9A9

^5^ECOSPHERE, Dept. of Biology, University of Antwerp, Groenenborgerlaaan 171, 2020 Antwerp, Belgium,

^6^Laboratory of Ecophysiology and Molecular Evolution, Brazilian National Institute for Research of the Amazon (INPA), Manaus, Brazil

**Address for Correspondence:** C.M. Wood, Dept. of Zoology, University of British Columbia, Vancouver, BC, Canada V6T 1Z4; phone**:** 1-604-827-1576; email: [woodcm@zoology.ubc.ca](mailto:woodcm@zoology.ubc.ca)

**ORCID IDs:**

Chris M. Wood: 0000-0002-9542-2219

Anne Crémazy: 0000-0002-0918-2336

Carolyn Morris: 0000-0002-7254-8433

Ora Johannsson 0000-0002-7944-379X

Gudrun De Boeck: 0000-0003-0941-3488

Adalberto Luis Val: 0000-0002-3823-3868

**Funding Information:** Supported by an NSERC (Canada) Discovery Grant (RGPIN-2023-03714) to CMW. AC was supported by an NSERC (Canada) Discovery Grant (RGPIN-2019-04400). This study was partially funded by CNPq (Brazilian National Research Council), CAPES (Coordination of Superior Level Staff Improvement), and FAPEAM (Amazonas State Research Foundation) via funding for INCT ADAPTA (CNPQ process N◦465540/2014-7, CAPES – Finance Code 001, and FAPEAM process 062.01187/2017) to ALV. ALV is recipient of a research fellowship from CNPq. CMW and GDB received ADAPTA Fellowships.

**Supplementary Table S1**

The results of two preliminary tests to see if the order of measurement affected the TEP values (in mV) recorded. In the first test (top panel), the fish was transferred into the control medium (INPA water, pH 7.0) and allowed to settle for 5 min prior to each of three measurement cycles in the control medium. In the second test (lower panels), 4 fish were transferred into the control medium (INPA water, pH 7.0) on the first cycle, and into the experimental medium (INPA water, pH 4.0) on the second cycle, while for 4 different fish, the order was reversed. Again, the fish was allowed to settle in the appropriate medium for 5 min prior to each measurement cycle. In each test, each TEP value is the mean for three measurements within each cycle for the same individual fish. In the first test, there were no significant differences (i.e. P >0.05) among the results from the three cycles, whereas in the second test, there was no significant difference (i.e. P >0.05) between the results from the two different cycle orders. Both tests indicated that the order of measurement did not matter.

1^st^ Cycle 2^nd^ Cycle 3^rd^ Cycle

Fish pH 7.0 pH 7.0 pH 7.0

A -18.1 -17.9 -25.0

B -18.4 -25.8 -24.6

C -22.4 -22.7 -21.7

D -37.1 -28.9 -29.5

F -30.0 -20.5 -25.5

Mean **-25.2 -23.2 -25.3**

SEM 3.7 1.9 1.3

_________________________________________________________________________________

1^st^ Cycle 2^nd^ Cycle 1^st^ Cycle 2^nd^ Cycle

Fish pH 7.0 pH 4.0 Fish pH 4.0 pH 7.0

M -26.0 -5.6 0 -0.8 -17.4

R -24.1 -9.8 p -10.0 -28.7

S -22.6 -8.4 Q -7.5 -22.3

T -29.3 -14.6 U -13.6 -32.0

**Mean** **-25.5 -9.6 Mean -8.0** **-25.1**

SEM 2.0 2.7 SEM -3.8 -4.6
